# Real time monitoring of *Staphylococcus aureus* biofilm sensitivity towards antibiotics with isothermal microcalorimetry

**DOI:** 10.1371/journal.pone.0260272

**Published:** 2022-02-16

**Authors:** Andi Rofian Sultan, Mehri Tavakol, Nicole A. Lemmens-den Toom, Peter D. Croughs, Nelianne J. Verkaik, Annelies Verbon, Willem J. B. van Wamel

**Affiliations:** 1 Department of Medical Microbiology and Infectious Diseases, Erasmus University Medical Center, Rotterdam, The Netherlands; 2 Department of Microbiology, Faculty of Medicine, Hasanuddin University, Makassar, Indonesia; Universidade Nova de Lisboa, PORTUGAL

## Abstract

Biofilm-associated infections with *Staphylococcus aureus* are difficult to treat even after administration of antibiotics that according to the standard susceptibility assays are effective. Currently, the assays used in the clinical laboratories to determine the sensitivity of *S*. *aureus* towards antibiotics are not representing the behaviour of biofilm-associated *S*. *aureus*, since these assays are performed on planktonic bacteria. In research settings, microcalorimetry has been used for antibiotic susceptibility studies. Therefore, in this study we investigated if we can use isothermal microcalorimetry to monitor the response of biofilm towards antibiotic treatment in real-time. We developed a reproducible method to generate biofilm in an isothermal microcalorimeter setup. Using this system, the sensitivity of 5 methicillin-sensitive *S*. *aureus* (MSSA) and 5 methicillin-resistant *S*. *aureus* (MRSA) strains from different genetic lineages were determined towards: flucloxacillin, cefuroxime, cefotaxime, gentamicin, rifampicin, vancomycin, levofloxacin, clindamycin, erythromycin, linezolid, fusidic acid, co-trimoxazole, and doxycycline. In contrast to conventional assays, our calorimetry-based biofilm susceptibility assay showed that *S*. *aureus* biofilms, regardless MSSA or MRSA, can survive the exposure to the maximum serum concentration of all tested antibiotics. The only treatment with a single antibiotic showing a significant reduction in biofilm survival was rifampicin, yet in 20% of the strains, emerging antibiotic resistance was observed. Furthermore, the combination of rifampicin with flucloxacillin, vancomycin or levofloxacin was able to prevent *S*. *aureus* biofilm from becoming resistant to rifampicin. Isothermal microcalorimetry allows real-time monitoring of the sensitivity of *S*. *aureus* biofilms towards antibiotics in a fast and reliable way.

## Introduction

*Staphylococcus aureus* is a notorious pathogen in post-surgery complications and severe infections as endocarditis, bacteremia and bone and joint infections [1, 2]. More than 70% of the cases of bone and joint infections are caused by biofilm-related *S*. *aureus* [3–7]. Biofilm-associated *S*. *aureus* infections are difficult to treat since the bacteria within the biofilm can be highly resistant to antibiotics and host immune responses [8–12].

Biofilms have been defined as aggregates of microorganisms in which bacterial cells are frequently embedded in a self-produced matrix of extracellular polymeric substances (EPS) that are adherent to each other and/or a surface [13–20]. The presence of an extracellular matrix that protects bacteria within the biofilm is one of the biofilm signatures that differentiates them from their planktonic form. The EPS matrix functions as a shield or repellent [21] to protect the bacteria from the immune system of the host like for instance antimicrobial peptides (AMP) and phagocytosis. The EPS matrix as a shield makes antibiotic therapy more difficult, leading to prolonged infections and more severe complications including bacteremia and death [22–25]. Despite the fact that bacteria from biofilms are different from their planktonic counterparts, the current antimicrobial susceptibility testing (AST) for *S*. *aureus* isolated from biofilm-related infections still uses planktonic bacteria. Unfortunately, this practice leads to overestimation of antibiotic effectivity as biofilm-associated bacteria show an increase tolerance towards antibiotics [26, 27]. Bacterial tolerance to antibiotics is intrinsic and mostly without any need for genetic alteration [26, 28]. The development of persister cells [29–31] and extracellular matrix formation [14–20] are among to the main causes of biofilm tolerance towards many antibiotic treatments.

The current assays to monitor antibiotic susceptibility of biofilms, such as standard plate counts, microtiter plate assay, and post-experimental staining, are labor intensive and have low reproducibility [32–36]. Furthermore, there are still questions marks regarding the applicability of these readout systems in a clinical setting [32, 34, 37–39] and most of all, these assays add an extra delay for obtaining results. Therefore, new strategies for monitoring biofilm tolerance in a quick and reproducible way are needed. Previously isothermal microcalorimetry has been studied for application in antimicrobial studies in a research setting [40–42] with encouraging observations. This technology allows to constantly determine the metabolism status of bacteria, with a threshold of approximately 1x10^4^ cells, by monitoring the heat-flow [40, 43] and detect any change in bacterial metabolic rate due to administration of drugs such as antibiotics [40]. Since it monitors bacterial metabolism instead of for instance colony forming unit (CFU), isothermal microcalorimetry can be applied to biofilm-associated bacteria, without having to disturb the biofilm itself.

In this study we describe the development of a reproducible method to generate *S*. *aureus* biofilms in an isothermal microcalorimeter setup and test the effectivity of several clinically relevant antibiotics [44–47] directly to these biofilms. Since previous studies show that bacteria in biofilm are extremely tolerant to antibiotics [32, 37, 48, 49], the effect of the maximum serum concentration of 13 commonly used antibiotics was studied in our calorimetry-based biofilm susceptibility test (CBST). In addition, these antibiotics were also assayed with current available antimicrobial susceptibility testing; VITEK^®^ 2 system and broth microdilution method. We managed to develop a fast and reproducible real-time method to monitor *S*. *aureus* biofilm sensitivity towards antibiotics for clinical application.

## Materials and methods

### Bacterial strains and growth condition

The *S*. *aureus* strains used in this study belong to the important genetic lineages found in humans [50] and are listed in Table 1. All strains were plated on Trypticase^TM^ Soy Agar (TSA) with 5% sheep blood overnight at 37°C (Becton Dickinson, Breda, The Netherlands). Presence of *mecA* gene, making the bacteria resistant to β-lactam antibiotics, was tested according to PCR protocol as described previously [51]

**Table 1:** Strains of *S*. *aureus* used.

Strain	Genetic Background	Description	Ref(s)
Mup15	CC15	MSSA, clinical isolate	[52]
Mup3199	CC25	MSSA, nasal isolate	[53]
Mup2723	CC30	MSSA, clinical isolate	[53]
Mup2396	CC45	MSSA, clinical isolate	[53]
Mup2704	ST72	MSSA, clinical isolate	[53]
MW2	CC1, USA400	MRSA, clinical isolate	[54]
Mu50	CC5	MRSA, clinical VISA isolate	[55]
SAC042W	CC8, USA300	MRSA, clinical isolate	[56]
M116	CC8, ST239	MRSA, clinical isolate	[12]
RWW146	CC398	MRSA	[57, 58]

### Antimicrobial susceptibility testing (AST)

Seven bactericidal and six bacteriostatic antibiotic drugs were selected for the experiments (Table 2). Susceptibility of all strains towards listed antibiotics were tested in VITEK^®^ 2 system (bioMérieux Benelux B.V, Zaltbommel, The Netherlands) according to manufacturer protocols.

**Table 2:** List of antibiotics.

Antibiotic	Class	Antibacterial potency	Maximum serum Concentration (μg/mL)	Ref(s)
Flucloxacillin (FLX)	Isoxazolyl penicillin	Bactericidal	16	[59]
Cefuroxime (CXM)	2^nd^ gen. cephalosporins	Bactericidal	8	[60]
Cefotaxime (CTX)	3^rd^ gen. cephalosporins	Bactericidal	16	[61]
Gentamicin (GEN)	Aminoglycosides	Bactericidal	16	[62]
Rifampicin (RIF)	Other	Bactericidal	8	[63]
Vancomycin (VAN)	Glycopeptides	Bactericidal	16	[64]
Levofloxacin (LVX)	Quinolones	Bactericidal	8	[65]
Clindamycin (CLI)	Other	Bacteriostatic	8	[66]
Erythromycin (ERY)	Macrolides	Bacteriostatic	8	[67]
Linezolid (LZD)	Other	Bacteriostatic	16	[68]
Fusidic acid (FD)	Other	Bacteriostatic	32	[69]
Co-trimoxazole (SXT)	Antifolate agents	Bacteriostatic	32	[70]
Doxycycline (DOX)	Tetracyclines	Bacteriostatic	4	[71]

To determine the minimal inhibitory concentration (MIC) of flucloxacillin, cefuroxime, cefotaxime, gentamicin, rifampicin, clindamycin, erythromycin, vancomycin, linezolid, levofloxacin, fusidic acid, co-trimoxazole, and doxycycline, broth microdilution (BMD) assay was performed on all strains according to the European Committee on Antimicrobial Susceptibility Testing (EUCAST). A hundred milliliter of 1:100 dilution of each strain (a 0.5 McFarland in NaCl 0.9%) in Mueller-Hinton II broth (MH II) (Oxoid, Hampshire, UK) was added to 100 μl MH II into a sterile round-bottom 96-well polystyrene tissue culture plate (Costar no. 3596; Corning Inc., Corning, N.Y.) containing serial dilutions of antibiotics. After 24 hours of incubation, the OD_600nm_ was read in a microplate reader (Epoch 2 Microplate reader, BioTek Instruments, Inc., Winooski, VT, USA). Interpretation of the results (Table 4) was done according to European Committee on Antimicrobial Susceptibility Testing (EUCAST) breakpoint tables for interpretation of MICs Version 11.0.

### Calorimetry-based biofilm susceptibility (CBS) assay

To determine biofilm fitness during co-incubation with antibiotics, we grew biofilms in an isothermal microcalorimetry set up according to previous protocol [11] with some modifications. Overnight culture of *S*. *aureus* strain on blood agar was suspended in 5 ml NaCl 0.9% until OD_600nm_ of 0.50 was reached, then 10 μl of it was mixed with 9990 μl of IMDM growth media to create a 1:1000 dilution. Ten microliters of this suspension were added into sterile flat-bottom calWell^TM^ insert or ampoule (CalScreener^TM^, SymCel, Spånga, Sweden) containing 190 μl IMDM. Plates were subsequently incubated for 1 hour under 150 rpm orbital shaking at 37°C to allow the bacteria to adhere. The adhered bacteria were then washed once and refreshed with 200 μL of fresh IMDM. These ampoules were inserted into sealed platinum tube before being placed inside of a multi-channel isothermal micro-calorimeter (calScreener^TM^, SymCel, Spånga, Sweden) for real-time measurement of heat-flow that is being emitted by the now created biofilm-associated *S*. *aureus* during 24 hours incubation at 37°C. After 24 hours of incubation, the biofilms were washed and refreshed once again with 200 μL new IMDM with or without desired antibiotics concentrations (Table 2) and then inserted back into the microcalorimeter to measure the heat-flow being produced by bacteria within the biofilms for another 24 hours. This multi-channel isothermal microcalorimeter is able to measure 32 samples simultaneously and the results are given as heat-flow versus time. To determine the sensitivity of the biofilm-associated cells towards antibiotics, the percentage of heat-flow of the treated biofilm relative to the untreated (control) were calculated, which is termed: biofilm fitness.

### Statistical analysis

Statistical analysis was performed by using the Prism 5.0 package (Graph Pad Software, San Diego, CA, USA) and Microsoft Excel 2010.We used unpaired t-test or one-way ANOVA for data analysis, where a two-sided P ≤ 0.05 was considered as statistically significant. All experiments were independently repeated for three times and the median with range was determined.

## Results

### Antimicrobial susceptibility testing of planktonic bacteria

Using VITEK^®^ 2 system and PCR for the presence of the *mecA* gene, we confirmed that 5 strains are MSSA and the other 5 are MRSA. AST results from VITEK^®^ 2 system showed that *S*. *aureus* CC5 Mu50 was resistant to almost all of the 13 tested antibiotics, except for linezolid, fusidic acid, and co-trimoxazole (Table 3), therefore it was used as non-susceptible reference strain.

**Table 3:** The results of VITEK2^®^ system for all strains toward tested antibiotics.

Strain		VITEK2^®^											
		OXA	FOX Screen	GEN	RIF	VAN	CIP	CLI	ERY	LZD	FD	SXT	TET
**MSSA**	**CC15**	0,5	-	≤0.5	≤0,03	1	≤0.5	0,25	1	2	≤0.5	≤10	≤1
	**CC25**	1	-	≤0.5	≤0,03	1	≤0.5	0,25	1	2	≤0.5	≤10	≥16
	**CC30**	≤0.25	-	≤0.5	≤0,03	≤0.5	≤0.5	0,25	0,5	2	≤0.5	≤10	≤1
	**CC45**	0,5	-	≤0.5	≤0,03	1	≤0.5	0,25	0,5	2	2	≤10	≤1
	**ST72**	0,5	-	≤0.5	≤0,03	1	≤0.5	0,25	1	2	≤0.5	≤10	≤1
**MRSA**	**CC1 (MW2)**	≥4	+	≤0.5	≤0,03	1	≤0.5	0,25	1	2	≤0.5	≤10	≤1
	**CC5 (Mu50)**	≥4	+	≥16	≥4	4	≥8	≥4	≥8	2	≤0.5	≤10	≥16
	**CC8**	≥4	+	≤0.5	≤0,03	1	≥8	0,25	1	2	≤0.5	≤10	≤1
	**ST239**	≥4	+	≥16	≤0,03	≤0.5	≥8	0,25	≥8	2	≤0.5	≥320	≥16
	**CC398**	≥4	+	≥16	≤0,03	≤0.5	≤0.5	≥4	≥8	2	≤0.5	80	≥16

*green = susceptible, red = resistant

Antibiotic susceptibility testing was also performed using a broth microdilution (BMD) susceptibility assay. The obtained data were in concordance with the VITEK^®^ results. The MICs of all strains toward the 13 tested antibiotics can be found in the Table 4.

**Table 4:** The results of broth microdilution susceptibility testing of all strains.

Strain		Minimal Inhibitory Concentration (MIC) μg/mL												
		Bactericidal							Bacteriostatic					
		FLX	CXM	CTX	GEN	RIF	VAN	LVX	CLI	ERY	LZD	FD	SXT	DOX
**MSSA**	**CC15**	0,25	2	4	0,5	0,0156	1	0,25	0,0625	1	4	0,5	1	0,25
	**CC25**	0,5	4	4	0,5	0,0078	1	0,125	0,125	1	4	0,25	1	4
	**CC30**	0,25	2	2	0,5	0,0078	1	0,25	0,125	0,5	4	0,25	1	0,25
	**CC45**	0,25	2	4	1	0,0312	1	0,25	0,125	1	4	4	1	0,5
	**ST72**	0,25	2	4	0,5	0,0078	1	0,25	0,0625	1	4	0,25	1	0,5
**MRSA**	**CC1 (MW2)**	8	256	256	0,5	0,0078	1	0,25	0,0625	1	2	0,125	1	0,5
	**CC5 (Mu50)**	640	>1024	>1024	256	>5000	4	16	>1000	>1024	2	0,25	1	8
	**CC8**	8	1024	128	0,5	0,0078	1	8	0,0625	1	4	0,25	1	0,25
	**ST239**	480	>1024	>1024	1024	0,0078	1	8	0,0625	>1024	4	0,25	625	8
	**CC398**	40	>1024	256	128	0,25	2	0,5	1000	>1024	2	0,5	2500	4

*green = susceptible and red = resistant

### Data interpretation of CBS assay

**Fig 1:**
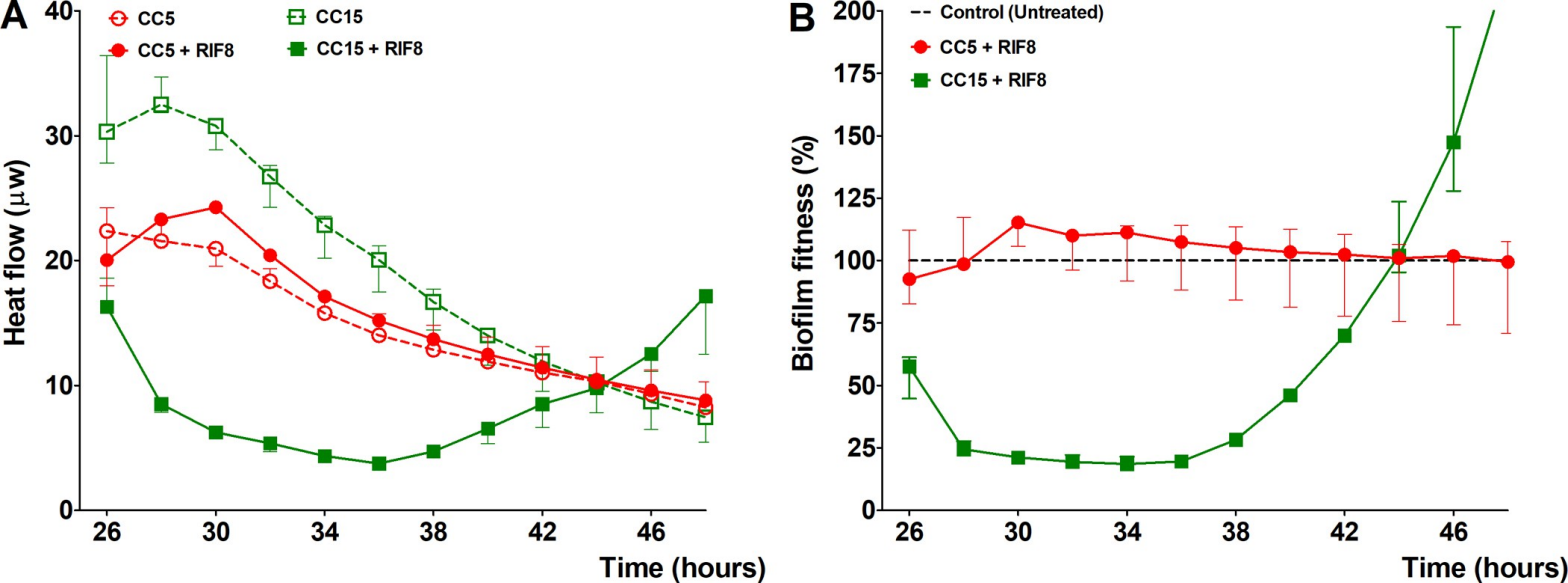
Normalization of data generated by isothermal microcalorimeter. Heat-flow of Mu50 (CC5) and CC15 during co-incubation with and without 8 μg/mL rifampicin (A). Biofilm fitness of Mu50 (CC5) and CC15 during co-incubation with and without 8 μg/mL rifampicin is given as percentage of the heat-flow of the rifampicin-treated biofilms relative to untreated biofilms (B). Dashed black lines indicate untreated (control) (B). Error bars represent median with range (n = 3).

For setting up the assay we started out by treating biofilms of *S*. *aureus* with the most active antibiotic against biofilms available: rifampicin. We are aware that rifampicin single therapy should not be used against biofilm-associated bacteria, as this often results in development of resistance [72–77]. So, 24 hour-old biofilms of *S*. *aureus* CC15 were treated with 8 μg/mL, Mu50 (CC5) was included as a rifampicin resistant control. All experiments were performed in triplicate and median values were calculated. Based on VITEK^®^ 2 and BMD analyses, we anticipated Mu50 (CC5) to be resistant to rifampicin which clearly can be seen in Fig 1A where the rifampicin-treated biofilms showed almost similar heat-flow signals as the untreated control. The rifampicin sensitive strain CC15 (according to VITEK^®^ 2 and BMD analyses), showed initially a steep incline-, followed by a 10 hours period of slow inclining -, finishing with a period of increasing heat-flow (Fig 1A). To determine the sensitivity of the biofilm-associated cells of these strains towards rifampicin in a more accessible way, we calculated the percentage of heat-flow of the treated biofilm relative to the untreated (control), which hereafter is termed: biofilm fitness (Fig 1B).

The biofilm fitness of Mu50 (CC5) is almost similar to the untreated control, approximately 100%, and remains the same during the whole co-incubation time, indicating that biofilms of Mu50 are resistant to the given concentrations of rifampicin (Fig 1B). The biofilm fitness of CC15 was approximately 75% reduced after exposure to rifampicin (8 μg/mL) and remains low for 10 hours where after it increases rapidly again. After 20 hours of co-incubation, the biofilm-associated CC15 bacteria were taken out of the calorimeter and subsequently tested with the VITEK^®^ 2 system, and as expected this initially rifampicin-sensitive strain had become resistant towards the antibiotic.

### Monitoring the effect of single antibiotic exposure

#### Rifampicin

**Fig 2:**
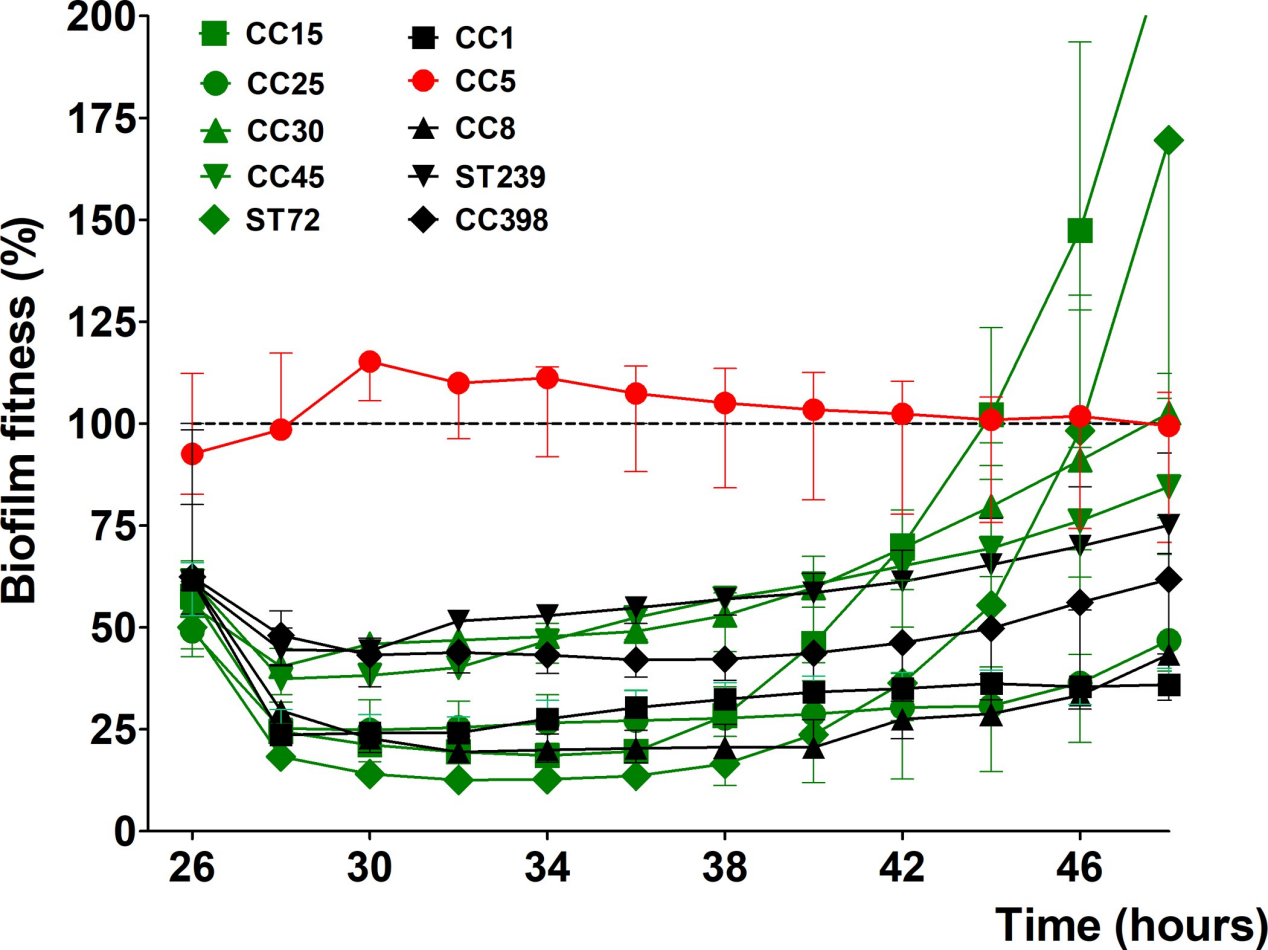
Sensitivity of biofilm-associated *S*. *aureus* strain to rifampicin (RIF). The 24 hours kinetic of biofilm fitness generated by MSSA and MRSA strains during co-incubation with 8 μg/mL rifampicin relative to untreated biofilm fitness. Mu50 (CC5) is used as non-susceptible control (red). Dashed horizontal lines indicate control (untreated biofilm). Error bars represent median with range (n = 3).

To further investigate the effect of rifampicin on biofilm-associated *S*. *aureus*, we assayed 4 additional MSSA and 4 MRSA strains representing the different genetic background found in humans. From VITEK^®^ 2 system (Table 3) and BMD data (Table 4), all strains, with the exception for the earlier mentioned Mu50 (CC5), were sensitive and showed an early reduction in biofilm fitness after administration 8 μg/mL of rifampicin (Fig 2). Within 24 hours of co-incubation, 5 out of 10 strains; CC15, CC30, CC45, ST72, and ST239 show an increase in their biofilm fitness, which might be an indication for the development of tolerance towards rifampicin (Fig 2). Furthermore, 2 strains; CC15 (already shown in Fig 1) and ST72 developed during the later stages a strong increase of biofilm fitness after 10 hours of co-incubation with rifampicin (Fig 2), which was confirmed also for ST72 by follow-up VITEK^®^ 2 analyses as being resistance towards the antibiotic.

#### Flucloxacillin

**Fig 3:**
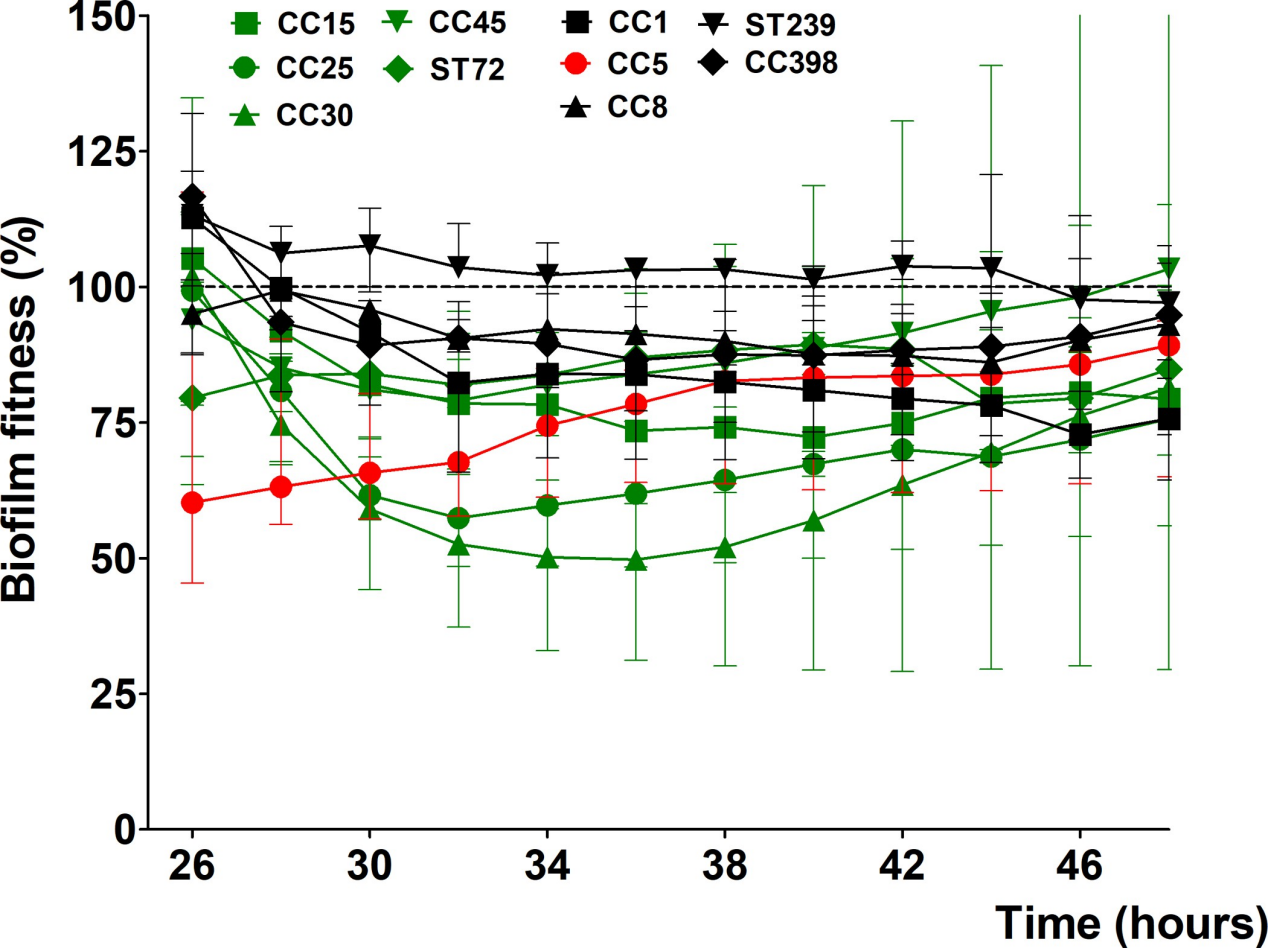
Biofilm-associated *S*. *aureus* sensitivity to flucloxacillin (FLX). The 24 hours kinetic of biofilm fitness of MSSA (CC15, CC25, CC30, CC45, and ST72) and MRSA (CC1, CC5, CC8, ST239, and CC398) strains towards 16 μg/mL flucloxacillin relative to untreated biofilm fitness. Mu50 (CC5) is used as non-susceptible control (red). Dashed horizontal lines indicate control (untreated biofilm). Error bars represent median with range (n = 3).

Twenty-four hour-old biofilms exposed to 16 μg/mL flucloxacillin showed that all MRSA strains were able to handle this maximum allowed serum concentration of the antibiotic (Fig 3), though there were some slight differences. For instance, ST239 seemed not to be affected by the exposure to flucloxacillin at all, yet Mu50 (CC5) despite having the highest MIC (Table 4) had the lowest biofilm fitness among all tested MRSAs (Fig 3).

The biofilms of the MSSA strains showed almost similar kinetic of their curve patterns as the MRSAs (Fig 3). Despite a quick decrease in biofilm fitness of CC25, CC30 and CC45 during exposure to flucloxacillin, the biofilm fitness of CC25 and CC45 bounced back after 6 hours, while CC30 show an increase of biofilm fitness after 12 hours of exposure to flucloxacillin (Fig 3). In the first 6 hours, the biofilm fitness of 3 out 5 of MSSA strains; CC15, CC45 and ST72 were found to be higher than Mu50 (CC5) (Fig 3). These data indicate that, despite the variable response of each strain to flucloxacillin in the beginning of exposure, during the 24 hours of incubation time, biofilms of all strains, independent if they are MRSA or not, can withstand flucloxacillin more or less equally.

#### Cephalosporin antibiotics

**Fig 4:**
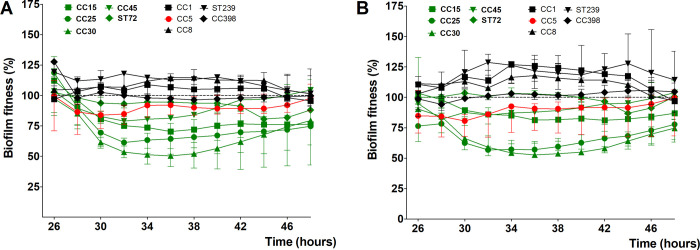
*Staphylococcus aureus* biofilm sensitivity to cephalosporins. The 24 hours kinetic of biofilm fitness of MSSA (CC15, CC25, CC30, CC45, and ST72) and MRSA (CC1, CC5, CC8, ST239, and CC398) strains towards 8 μg/mL cefuroxime (CXM) (A) and 16 μg/mL cefotaxime (CTX) (B) relative to untreated biofilm fitness. In both figures Mu50 (CC5) was used as non-susceptible control. Dashed horizontal lines indicate control (untreated biofilm). Error bars represent median with range (n = 3).

Administration of the other beta lactam antibiotics cefuroxime and cefotaxime show that biofilm fitness of the MSSA strains, except for ST72 strain, were more or equally sensitive toward cefuroxime (Fig 4A) and cefotaxime (Fig 4B) in comparison to Mu50 (CC5), though all bacteria were able to survive the exposure. On the contrary, in comparison to Mu50 (CC5) the other MRSA strains could withstand both antibiotics better. (Fig 4).

#### Vancomycin

**Fig 5:**
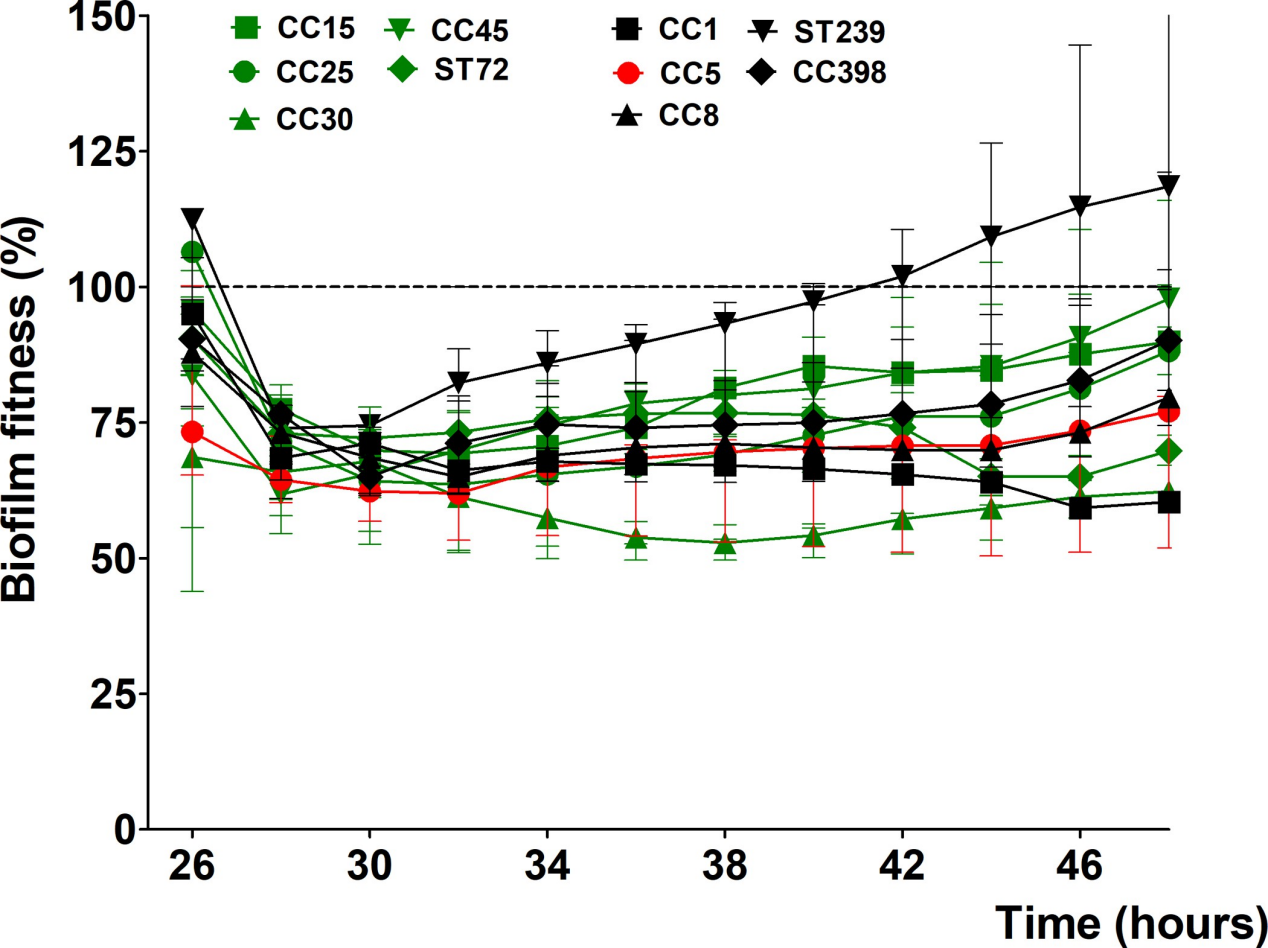
Biofilm-associated *S*. *aureus* sensitivity to vancomycin (VAN). The 24 hours kinetic of biofilm fitness of MSSA (CC15, CC25, CC30, CC45, and ST72) and MRSA (CC1, CC5, CC8, ST239, and CC398) strain towards 16 μg/mL vancomycin relative to untreated biofilm fitness. VISA strain Mu50 (CC5) was plotted in red. Dashed horizontal lines indicate control (untreated biofilm). Error bars represent median with range (n = 3).

When we assayed the MSSA and MRSA strains for sensitivity towards vancomycin 16 μg/mL, we observed a decrease in biofilm fitness for all strains within the first 2 hours of coincubation time. Three MSSAs (CC15, CC25, and CC45) and four MRSAs (CC5, CC8, ST239, and CC398) showed a steady recovery of biofilm fitness during exposure to vancomycin which was not observed for the other strains (Fig 5). Although Mu50 (CC5), based on broth microdilution and VITEK® 2 system, is considered to be a vancomycin intermediate-resistant *S*. *aureus* (VISA), biofilms of this strain were during the first 6 hours of exposure to vancomycin, among the most affected (Fig 5). Furthermore, similar to flucloxacillin and the cephalosporin exposure, no significant differences in sensitivity to vancomycin were found between biofilms of MSSA and MRSA strains.

#### Other antibiotics

**Fig 6:**
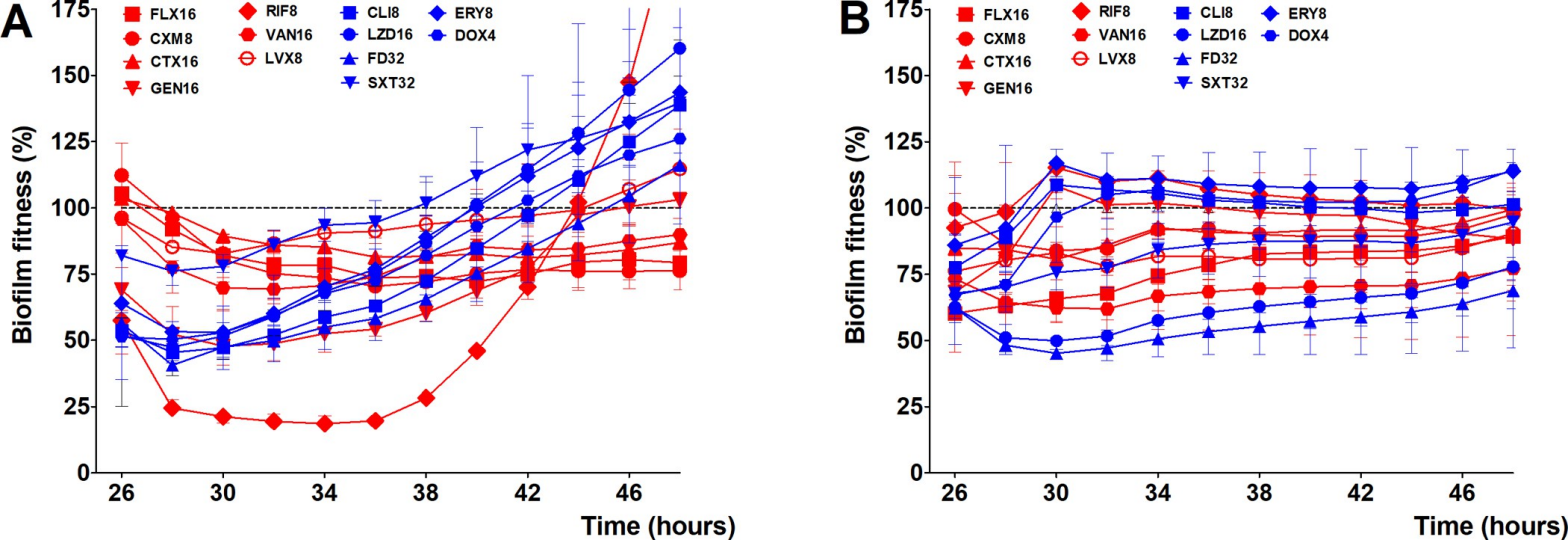
*Staphylococcus aureus* biofilm sensitivity to other antibiotics. The 24 hours kinetic of biofilm fitness of MSSA CC15 (A) and MRSA Mu50 (CC5) (B) strains co-incubated with maximum serum concentration of several bactericide (red) and bacteriostatic (blue) antibiotics relative to untreated biofilm fitness. Dashed horizontal lines indicate control (untreated biofilm). Error bars represent median with range (n = 3).

Since both beta-lactams and vancomycin were unable to reduce biofilm fitness of the strains studied after more than 24 hours of exposure, we studied the effect of the bactericidal antibiotic gentamicin and levofloxacin, and also bacteriostatic antibiotics clindamycin, erythromycin, linezolid, fusidic acid, co-trimoxazole, and doxycycline on *S*. *aureus* biofilms, and included previously mentioned data as well. Comparing the most sensitive MSSA (CC15) with the most resistant MRSA (Mu50 (CC5)), several things drew our attention. When we look at the data of Mu50 (CC5), the biofilm fitness during co-incubation with both the bacteriostatic and bactericidal antibiotics remains more or less the same (Fig 6B). As Mu50 (CC5) was found to be resistant to most antibiotics when grown in suspension (planktonic) (Tables 3 and 4), our in vitro microcalorimeter measurement on single antibiotic administration indicate that AST data from VITEK^®^ 2 system or broth microdilution assay for this resistant strain can be extrapolated to biofilms as well. For MSSA CC15 (Fig 6A), the reduction of biofilm fitness as a consequence of exposure to the various bactericide and bacteriostatic antibiotics was less than for rifampicin, despite the early reduction of its biofilm fitness in the first couple of hours of the exposure time. Interestingly, for the bactericidal antibiotics, in most cases the biofilm fitness remains the same throughout the exposure time, indicating that despite being sensitive to these antibiotics in planktonic state, biofilm of MSSA CC15 strain can withstand bactericidal antibiotics just like the biofilms of the MRSA strain CC5 (Fig 6). When we look at the bacteriostatic antibiotics, the biofilm fitness of MSSA CC15 after an initial reduction, in time increases in most of the cases linearly (Fig 6A). This phenomenon indicates a time dependent recovery of biofilm fitness when exposed to bacteriostatic antibiotics (Fig 6A).

Looking at the rest of the strains (S1 and S2 Figs), all MSSA strains show a similar pattern for the bacteriostatic antibiotics as CC15, with an initial reduction of the biofilm fitness followed by a more or less linearly increase of biofilm fitness during the rest of co-incubation time (S1 Fig). Dependent on the strains, MSSA strains show almost no difference (CC15 and ST72) or a moderate increase (CC25, CC30, and CC45) of biofilm fitness during co-incubation with a bactericidal antibiotic (S1 Fig). The biofilm fitness of both MRSA and MSSA strains exposed to bacteriostatic or bactericidal antibiotic show similar kinetics (S1 and S2 Figs) unless they were found to be resistant in the VITEK^®^ or in the broth microdilution assays. In the later cases biofilm fitness is almost not affected by antibiotic exposure.

### Monitoring the effect of combined antibiotics exposure

Since a single regimen of rifampicin could initially reduce biofilm fitness better than any other tested antibiotics, we looked for antibiotic combinations that could prevent the development of rifampicin resistant biofilms. As before, microcalorimetry was used to monitor the response of MSSA CC15 and ST72 biofilms during co-incubation with flucloxacillin, vancomycin, levofloxacin, and clindamycin in combination with rifampicin.

**Fig 7:**
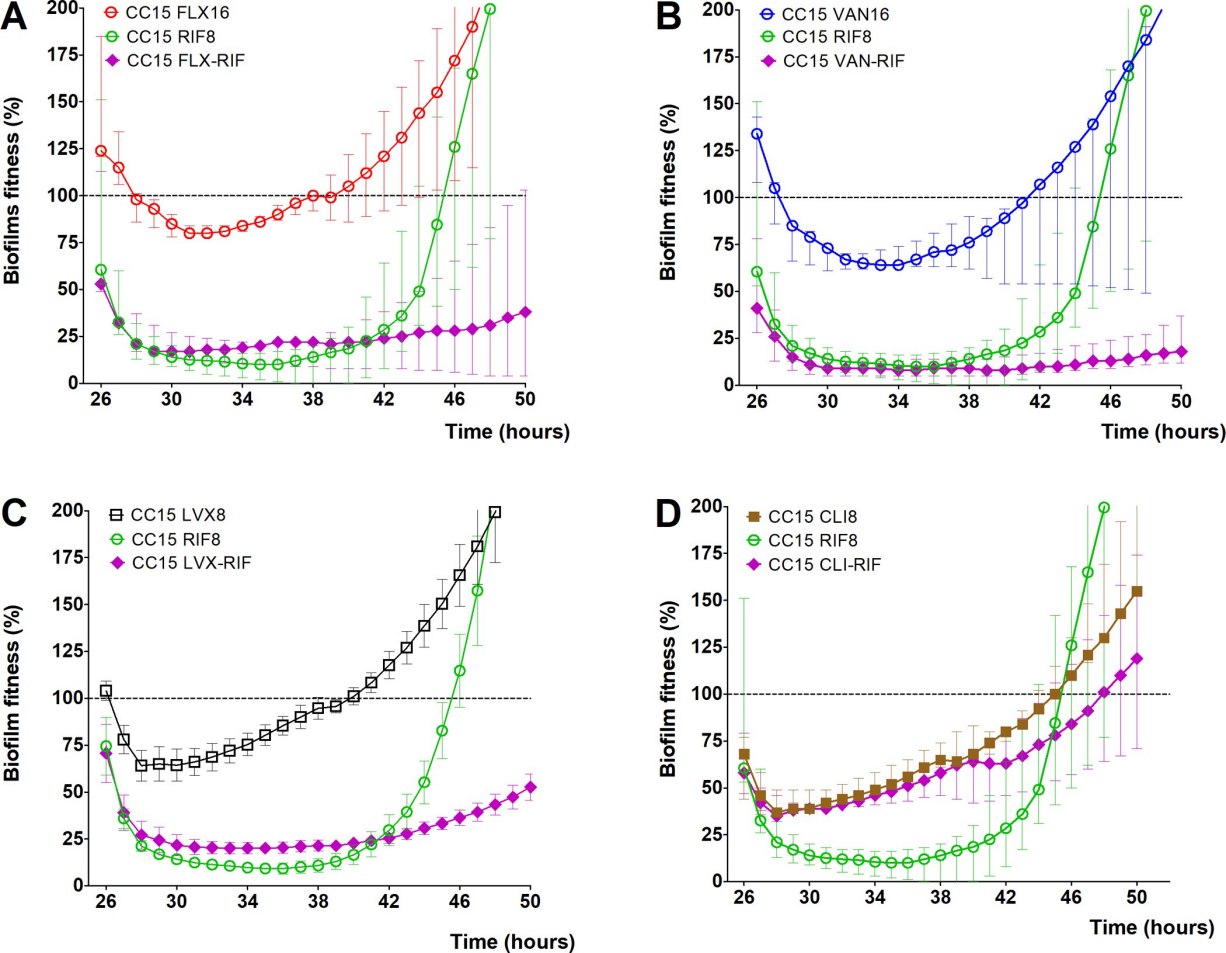
Biofilm-associated *S*. *aureus* sensitivity to combination antibiotics. The 24 hours kinetic of biofilm fitness of MSSA CC15 strain towards maximum serum concentration of flucloxacillin (FLX) (A), vancomycin (VAN) (B), levofloxacin (LVX) (C), and clindamycin (CLI) (D) in combination with 8 μg/mL rifampicin (RIF) was administered to 24 hour-old biofilms grown relative to untreated biofilm fitness. Dashed horizontal lines indicate control (untreated biofilm). Error bars represent median with range (n = 3).

Combining 8 μg/mL of rifampicin with either 16 μg/mL flucloxacillin, 16 μg/mL vancomycin or 8 μg/mL levofloxacin shows that the biofilm fitness remained low in both CC15 (Fig 7A–7C) and ST72 (S3A–S3C Fig). VITEK^®^ results analyses of the bacteria that were double treated, or treated only with rifampicin, flucloxacillin, vancomycin, or levofloxacin indicated that only the rifampicin single treated biofilms developed resistance. Interestingly, a different phenomenon was seen when rifampicin was combined with clindamycin. The combination of these antibiotics showed an antagonistic effect. Addition of 8 μg/mL clindamycin seemed to inhibit the effectiveness of rifampicin and prevented reduction of biofilm fitness of both CC15 (Fig 7D) and ST72 strain (S3D Fig). None of the single antibiotic regimens, including rifampicin alone, could kill the biofilm-associated bacteria but combination of rifampicin with other antibiotics such as vancomycin was able to reduce bacterial fitness and viability.

## Discussion

Using isothermal microcalorimetry, we developed a highly reproducible and sensitive assay to study the sensitivity of biofilm associated *S*. *aureus* cells in real time. When we look at biofilm sensitivity towards beta-lactams like flucloxacillin, cefuroxime and cefotaxime, we observed that both biofilms of MSSA and MRSA can withstand the maximal serum concentration of these antibiotics rather well. In our experimental setup, treatment of biofilm-associated *S*. *aureus* with the maximal serum value of vancomycin did not show any added value, most strains even outperformed the VISA Strain Mu50. As expected, exposure of MRSA biofilms to beta lactams, leads to very limited or no effect on biofilm fitness. On the contrary, MSSA biofilms exposed to gentamicin, levofloxacin, clindamycin, erythromycin, linezolid, fusidic acid, co-trimoxazole, or doxycycline generally led to an initial reduction then followed by a linear recovery of the biofilm fitness, which was seen more prominent in the bacteriostatic antibiotics. MRSA strains that were sensitive (based on VITEK® or broth microdilution data) to one of these antibiotics showed similar patterns as the MSSA strains.

Rifampicin was the most effective antibiotic during the first 12 hours of antibiotic exposure, yet as was expected [78], resistance was found in some strains. This finding supports the fact that rifampicin should not be used as monotherapy in the clinic [44, 75–77, 79]. Furthermore, although in general a significant reduction in biofilm fitness was found for most strains, in none of the cases rifampicin was able to kill all biofilm-associated bacteria. Combining rifampicin with flucloxacillin, vancomycin, or levofloxacin prevented the development of resistance during the time-course of these experiments. Future analyses are needed to determine whether long term treatment with these antibiotic combinations, also can prevent the development of rifampicin resistance. Of further interest is the observation that rifampicin in combination with vancomycin, can kill biofilm-associated *S*. *aureus*. In support to this finding, previously Niska, et al [80] showed in a *S*. *aureus* murine bone infection model that the combination of vancomycin with rifampicin was able to decrease the bacterial load in bone though the mechanism of this synergistic effect remains unclear [75, 80]. On the contrary combining rifampicin with clindamycin, neutralized the sensitivity of biofilm associated bacteria toward rifampicin.

As to be expected, when a strain was found to be resistant to an antibiotic using VITEK^®^ or the broth microdilution assay, these data could be extrapolated to biofilms. On the contrary, data from these antimicrobial susceptibility tests did not have any predictive value for biofilm-associated bacteria when the strains were found to be sensitive. These techniques test antibiotics on planktonic bacteria instead of biofilm associated ones, but with our calorimetry-based assay we were able to assay antibiotics directly to biofilm with high reproducibility. We realized that once persister cells develop, we presumably will not be able to detect them with isothermal microcalorimetry due to their low metabolism rates. Therefore, we always plated the bacteria when antibiotic treatment reduced the biofilm fitness to less than 10%.

In summary, here we showed that isothermal microcalorimetry can be used to monitor biofilm-associated bacteria sensitivity towards antibiotics in real time. With this calorimetry-based biofilm susceptibility assay, we demonstrated that bacteria within a biofilm can handle the maximum dose of antibiotics that can (safely) be reached in human serum. Furthermore, we were able to monitor the development of tolerance or resistance towards the applied antibiotics in real time. In a follow-up study, we anticipate to generate a mathematic model to quantify the development of tolerance and or resistance of biofilm associated cells to the administered antibiotics.

## Supporting information

S1 Fig *Staphylococcus aureus* biofilms of MSSA strains sensitivity to various antibiotics. The 24 hours kinetic of biofilm fitness of MSSA CC25 (A), CC30 (B), CC45 (C), and ST72 (D) strains co-incubated with maximum serum concentration of several bactericide (red) and bacteriostatic (blue) relative to untreated biofilm fitness. Dashed horizontal lines indicate control (untreated biofilm). Error bars represent median with range (n = 3).
(TIF)

S2 Fig *Staphylococcus aureus* biofilm of MRSA strains sensitivity to various antibiotics. The 24 hours kinetic of biofilm fitness of MRSA CC1 (A), CC8 (B), ST239 (C), and CC398 (D) co-incubated with maximum serum concentration of several bactericide (red) and bacteriostatic (blue) antibiotics relative to untreated biofilm fitness. Dashed horizontal lines indicate control (untreated biofilm). Error bars represent median with range (n = 3).
(TIF)

S3 Fig Biofilm-associated *S*. *aureus* sensitivity to combination antibiotics. The 24 hours kinetic of biofilm fitness of MSSA ST72 strain co-incubated with maximum serum concentration of flucloxacillin (FLX) (A), vancomycin (VAN) (B), levofloxacin (LVX) (C), and clindamycin (CLI) (D) in combination with 8 μg/mL rifampicin (RIF) relative to untreated biofilm fitness. Dashed horizontal lines indicate control (untreated biofilm). Error bars represent median with range (n = 3).
(TIF)
