# Supplementary figures and images for: Real time monitoring of Staphylococcus aureus biofilm sensitivity towards antibiotics with isothermal microcalorimetry
Source: PLoS One. 2022 Feb 16;17(2):e0260272. doi: 10.1371/journal.pone.0260272 (PMC8849495; doi:10.1371/journal.pone.0260272)

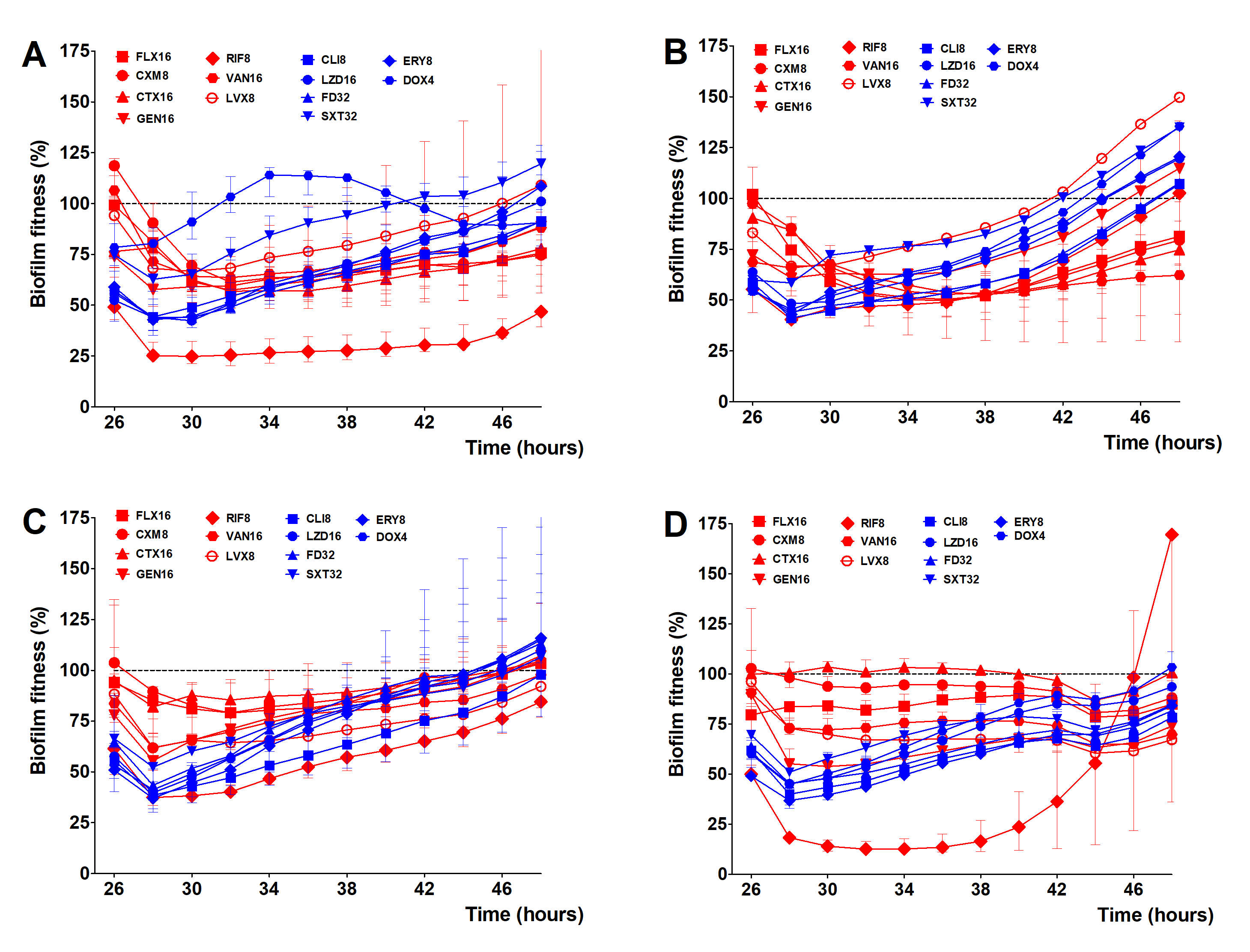

Supplement: S1 Fig — The 24 hours kinetic of biofilm fitness of MSSA CC25 (A), CC30 (B), CC45 (C), and ST72 (D) strains co-incubated with maximum serum concentration of several bactericide (red) and bacteriostatic (blue) relative to untreated biofilm fitness. Dashed horizontal lines indicate control (untreated biofilm). Error bars represent median with range (n = 3). (TIF) [file pone.0260272.s001.tif]

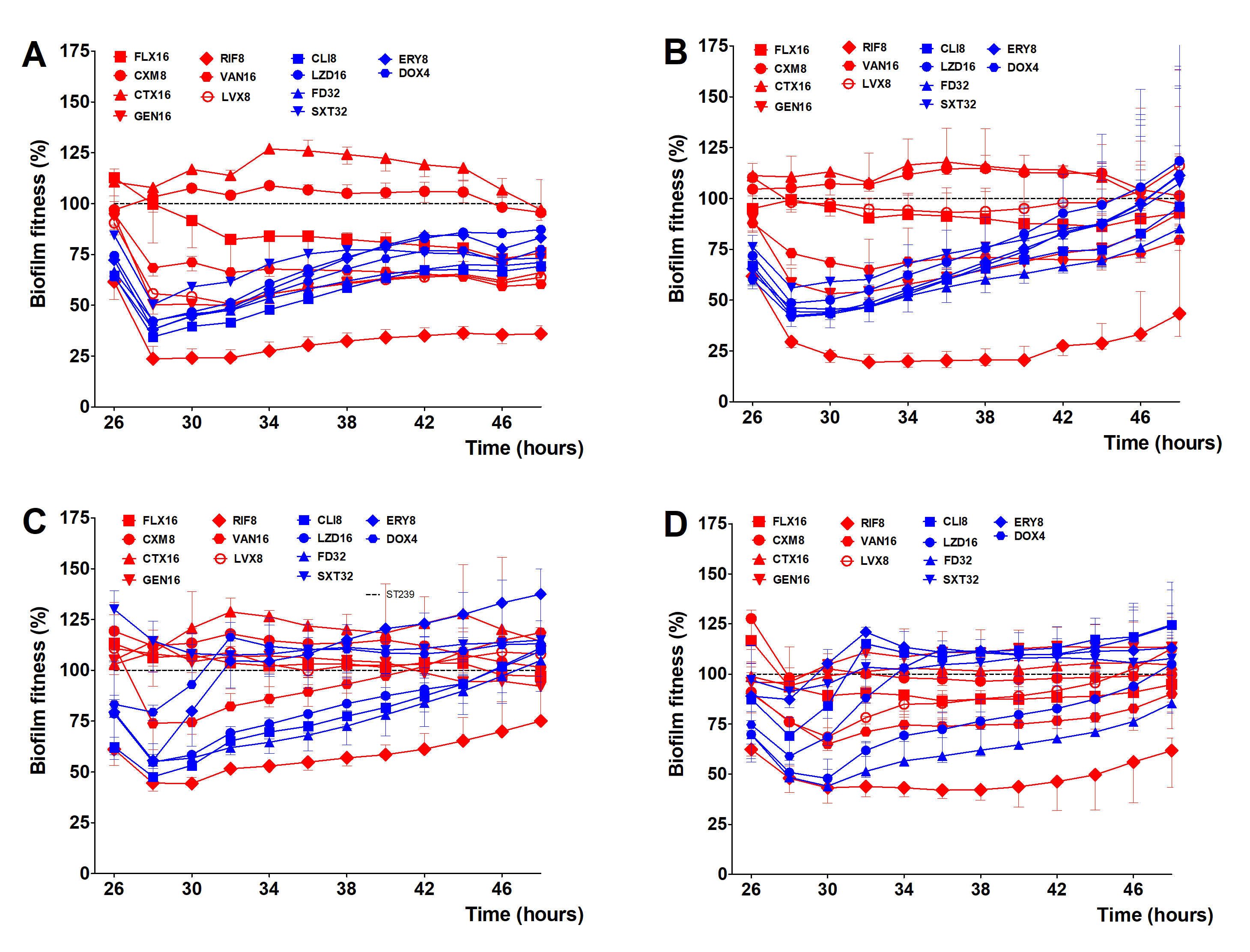

Supplement: S2 Fig — The 24 hours kinetic of biofilm fitness of MRSA CC1 (A), CC8 (B), ST239 (C), and CC398 (D) co-incubated with maximum serum concentration of several bactericide (red) and bacteriostatic (blue) antibiotics relative to untreated biofilm fitness. Dashed horizontal lines indicate control (untreated biofilm). Error bars represent median with range (n = 3). (TIF) [file pone.0260272.s002.tif]

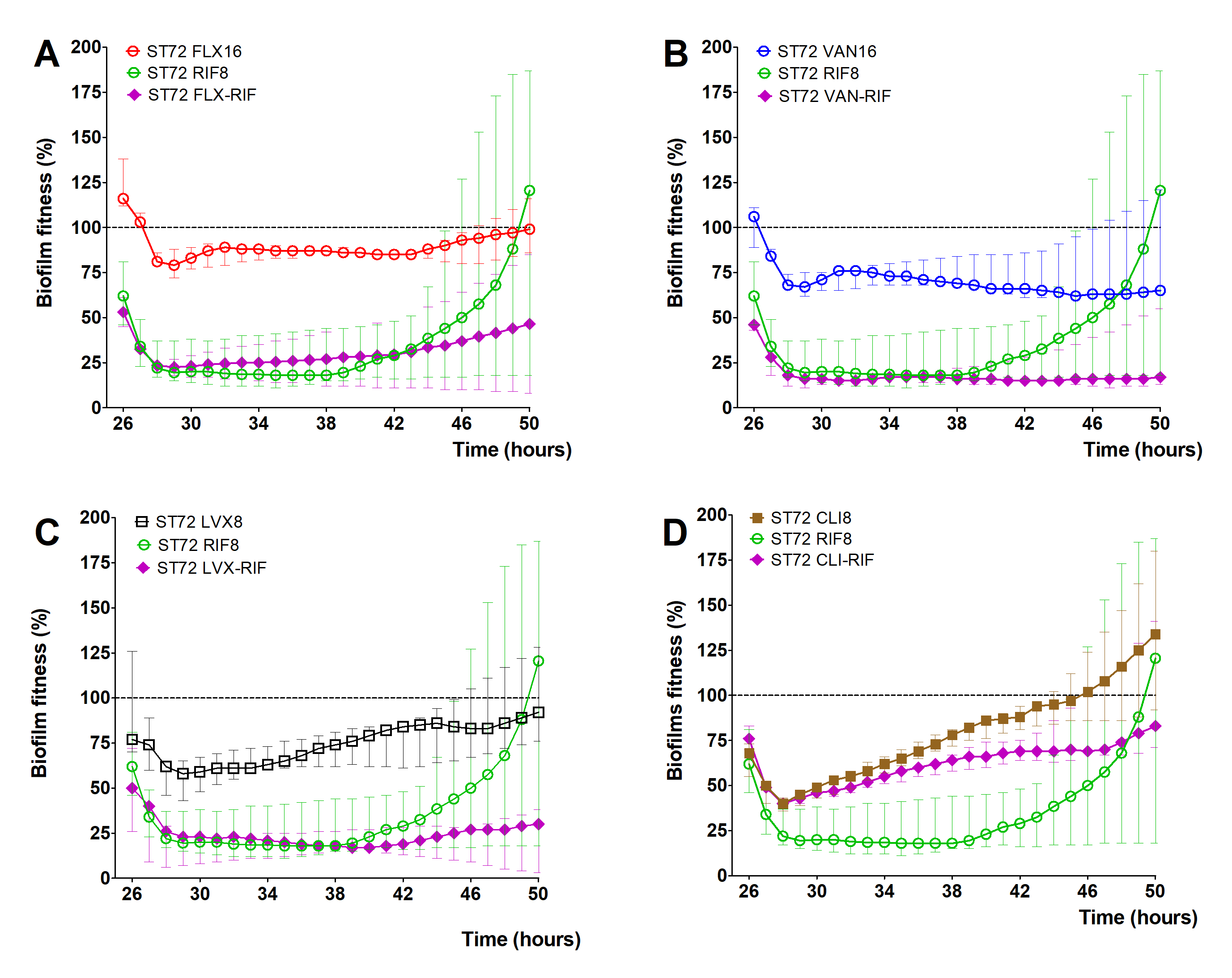

Supplement: S3 Fig — The 24 hours kinetic of biofilm fitness of MSSA ST72 strain co-incubated with maximum serum concentration of flucloxacillin (FLX) (A), vancomycin (VAN) (B), levofloxacin (LVX) (C), and clindamycin (CLI) (D) in combination with 8 μg/mL rifampicin (RIF) relative to untreated biofilm fitness. Dashed horizontal lines indicate control (untreated biofilm). Error bars represent median with range (n = 3). (TIF) [file pone.0260272.s003.tif]
